# Role of glutamine and its metabolite ammonia in crosstalk of cancer-associated fibroblasts and cancer cells

**DOI:** 10.1186/s12935-021-02121-5

**Published:** 2021-09-09

**Authors:** Xiao Li, Hongming Zhu, Weixuan Sun, Xingru Yang, Qing Nie, Xuedong Fang

**Affiliations:** 1 Department of Gastrointestinal Colorectal and Anal Surgery, China-Japan Union Hospital of Jilin University, Changchun, Jilin People’s Republic of China; 2 Department of Obstetrics and Gynecology, Second Hospital of Jilin University, Changchun, Jilin People’s Republic of China; 3 Department of Cardiology, Second Hospital of Jilin University, Changchun, Jilin People’s Republic of China

**Keywords:** Cancer-associated fibroblasts, Glutamine, Ammonia, Cancer cells, Tumor microenvironment

## Abstract

Cancer-associated fibroblasts (CAFs), the most abundant cells in the tumor microenvironment, play an indispensable role in cancer initiation, progression, metastasis, and metabolism. The limitations of traditional treatments can be partly attributed to the lack of understanding of the role of the tumor stroma. For this reason, CAF targeting is gradually gaining attention, and many studies are trying to overcome the limitations of tumor treatment with CAF as a breakthrough. Glutamine (GLN) has been called a “nitrogen reservoir” for cancer cells because of its role in supporting anabolic processes such as fuel proliferation and nucleotide synthesis, but ammonia is a byproduct of the metabolism of GLN and other nitrogenous compounds. Moreover, in some studies, GLN has been reported as a fundamental nitrogen source that can support tumor biomass. In this review, we discuss the latest findings on the role of GLN and ammonia in the crosstalk between CAFs and cancer cells as well as the potential therapeutic implications of nitrogen metabolism.

## Introduction

Glutamine (GLN) is a non-essential amino acid that can be synthesized by cells via GLN synthetase (GS). It is abundant in the blood in the form of free amino acids. Cancer cells absorb and utilize GLN at high rates [1, 2]. Previous studies have shown that GLN mainly functions as a supplement to TCA cycle and nucleotide biosynthesis [3, 4]. However, GLN plays an important role in all aspects of carcinogenesis, such as antioxidant defense, chromatin modification/gene transcription, bioenergy, transportation of other amino acids across the plasma membrane, and the regulation of cell signaling [5]. GLN metabolism-related inhibitors have been approved by the FDA for the treatment of cancer and other diseases, and many of these inhibitors are currently being investigated in clinical trials [6].

When GLN is broken down to glutamate, ammonia is formed as a byproduct and is generally considered a toxic metabolite. However, recent studies on the role of ammonia in tumors have started reporting diverging data; one hypothesis is that ammonia is a metabolic waste product that inhibit tumor growth, whereas the other is that an appropriate amount of ammonia can promote tumor growth and support biomass production [7, 8].

**Fig. 1:**
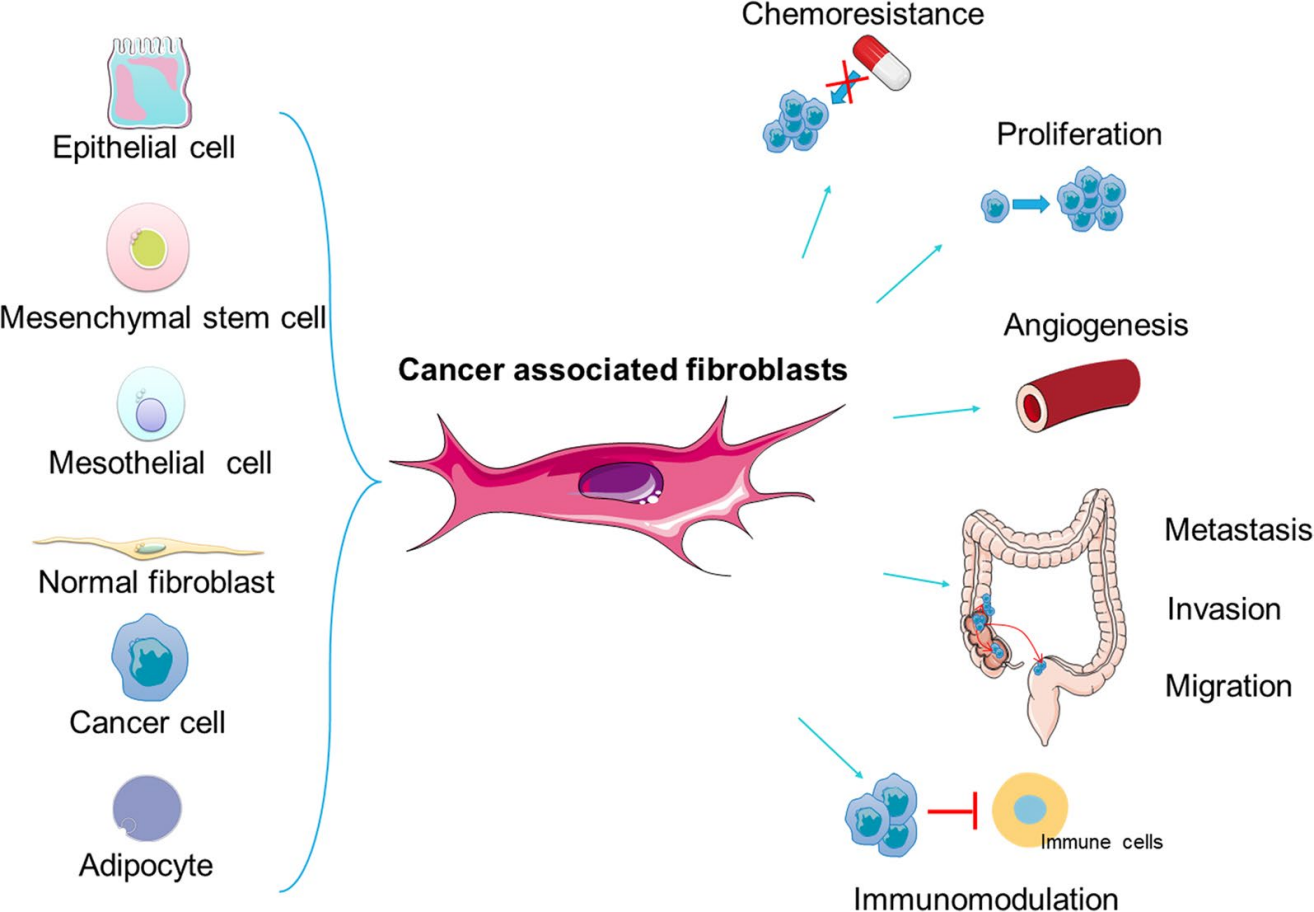
Origins and functions of CAFs. The main origin of CAFs include epithelial cells, mesenchymal stem cells, mesothelial cells, normal fibroblasts, cancer cells, adipocytes and so on. The influence of CAFs on tumor cells includes various aspects like drug resistance, tumor proliferation, metastasis, invasion, migration, immunomodulation, and angiogenesis

The tumor microenvironment (TME) plays an important role in the utilization of GLN and ammonia, synthesis and secretion of GLN, sensing of changes in the concentration of GLN and ammonia, secretion of cytokines that affect GLN metabolism in cancer cells, and alterations in tumor cell proliferation and metastasis [9]. The TME is a complex combination of both pro-tumorigenic and anti-tumorigenic components, including varieties of immune cells, endothelial cells, mesenchymal stem cells, and fibroblasts (both CAFs and normal fibroblasts), as well as the extracellular matrix (ECM). The imbalance between ECM synthesis and turnover results in vascular dysplasia, disordered collagen formation, and abnormal secretion of cytokines [10–13]. Data on these elements pave the way for further research on the crosstalk between cancer cells and cells located in the stroma. CAFs are the main producers of ECM and paracrine signals that promote the formation of stem cell niches, tumor growth, immunosuppression, metastasis, and chemoresistance (Fig. 1) [14].

GLN level in the ECM is regulated by both CAFs and other cancer cells [15]. The concentration and metabolites of GLN, particularly ammonia, act as signals that mediate the metabolic processes of cancer cells and CAFs [16]. GLN plays a vital role in tumor development because most tumor cells require GLN for growth. However, in the traditional sense, ammonia, a metabolic waste product, has also been reported to be conducive to tumor cell activity [7]. This new finding was possibly because the recent research on tumors is not limited to cancer cells alone but instead focuses on the complex and diverse TME [17]. Therefore, while reviewing the metabolism of GLN and ammonia, this article will focus on the metabolic processes in which fibroblasts participate, providing insights into the development of new treatment modalities for tumors.

## Role of GLN in cancer cells

Although most cancer metabolic pathways are associated with aerobic glycolysis [18], GLN, as a non-essential amino acid, also has a cancer-promoting effect in many tumors [19]. In some cancers, such as pancreatic and breast cancers, GLN is one of the most rapidly utilized nutrients after glucose [20, 21]. Davidson et al. reported that both lung cancer cells and normal lung cells utilize minute quantities of exogenous GLN [22]. Different levels of GLN are utilized in different cancers [22–24]. In hyperglutamine-dependent cancer cells, GLN is used to synthesize amino acids, proteins, lipids, and nucleotides and regulate cancer cell metabolism [25]. Here, we discuss the role of GLN in cancer cells as well as the mechanism underlying its metabolic regulation.

### GLN transport

**Fig. 2:**
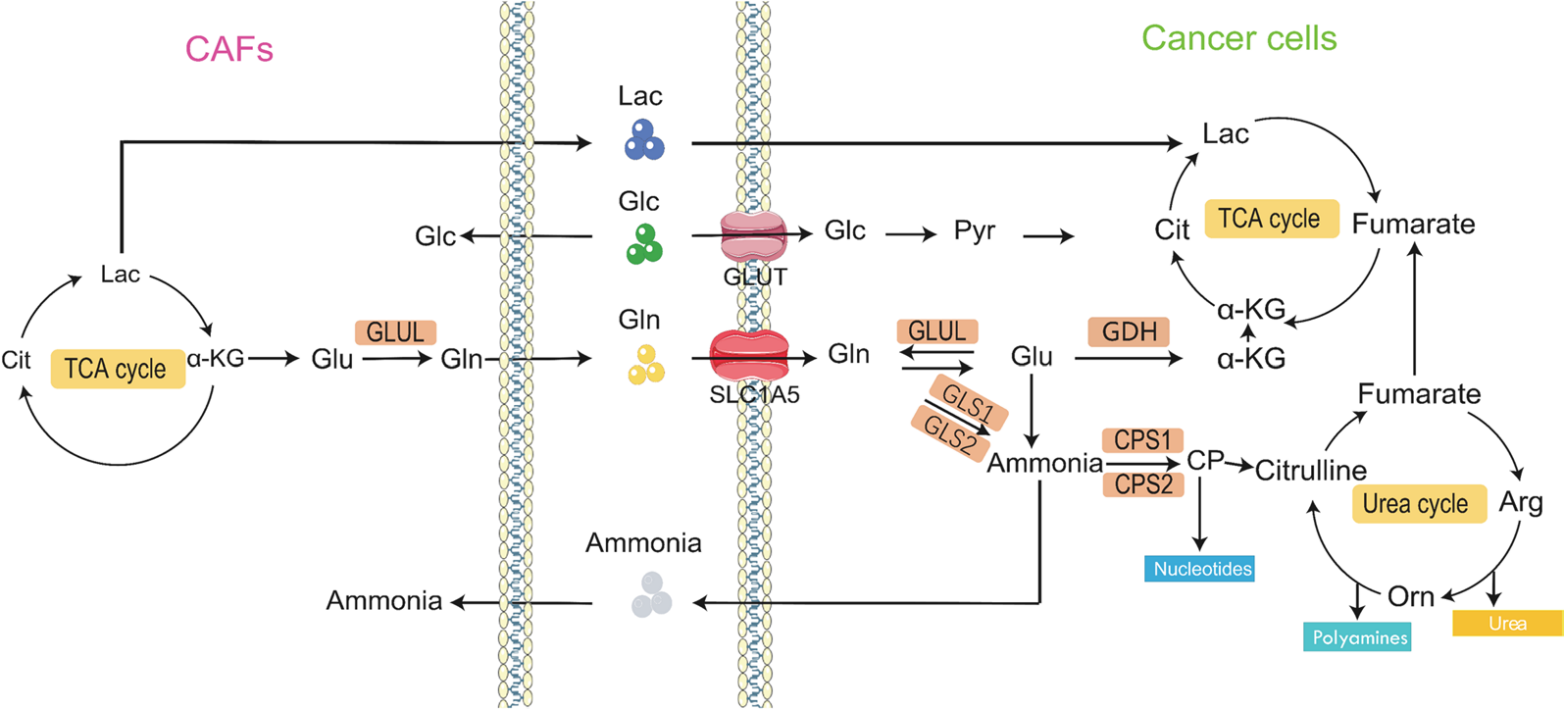
Glutamine metabolism between CAFs and cancer cells. CAFs can provide cancer cells with Lac, Gln and other nutrients into TCA cycle and urea cycle, and then promote the growth and development of tumor cells.The black pointed arrow heads indicate material transportation. The blue blunt arrowheads indicate inhibitory interactions. *Arg* ariginine, *α-KG* α-Ketoglutaric acid, *Cit* citrate, *Lac* lactose, *Glc* glucose, *Glu* glutamate, *GSH* glutathione, *Orn* ornithine, *PYR* pyruvate

Usually, GLN is transported into cancer cells mostly by alanine-serine-cysteine-transporter-2 (also called SLC1A5) followed by SLC6A14 and SLC38A5 [26]. According to the Cancer Cell Line Encyclopedia project and other studies, SLC1A5 is overexpressed in many solid cancers, such as colon, gastric, lung, and prostate cancers [27]. SLC1A5 is mainly involved in GLN import into cells, leading to cancer cell proliferation [28]. c-MYC, RNF5, and microRNA-137 have been confirmed to regulate SLC1A5 expression, but other mechanisms regulating SLC1A5 still need to be investigated [29–31]. GLN transported into cells via SLC1A5 can be directly used in cell metabolism or can be exchanged for leucine, valine, and other essential amino acids via the L-type amino acid transporter (also called SLC7A5) for indirect utilization in cells [32]. SLC7A5 is an external transporter of GLN that regulates the balance of the GLN flux [33]. GLN, when used in cell metabolism, can be catalyzed to glutamate via mitochondrial glutaminase (GLS), cytosolic enzymes, and other glutamate-producing enzymes. Glutamate may also be transported out of the cell by the SLC7A11 antiporter in exchange for cysteine [34]. SLC7A11 mainly regulates the redox status, ferroptosis, and intercellular signaling, leading to an increase in resistance to treatments such as chemotherapy and radiotherapy [35]. SLC7A11 expression is promoted by many oncogenes, such as *KRAS* and *PTEN* [36, 37]. Tumor suppressors, such as P53 activating transcription factor 3 and signal transducer and activator of transcription 3, inhibit SLC7A11 expression and transcription (Fig. 2) [38–40].

### Oncogenic regulation of GLN metabolism

Advances in research and deeper understanding of tumor have shown that the metabolism of tumor cells is regulated by various genes. Recent studies have found that *Myc,* *mTOR,* *p53,* and mitogen-activated protein kinase (*MAPK*) are the major oncogenes associated with GLN metabolism [41].

MYC is a proto-oncoprotein that regulates the uptake, utilization, and decomposition of GLN [42]. In some types of human and mouse cancers, MYC induces the overexpression of glutamate ammonia ligase (GLUL), also called GS, which is responsible for de novo GLN synthesis. Cell and animal experiments in mammary epithelial cancer, pancreatic ductal neoplasia, T-cell lymphoma, and lung cancer cells have reported that MYC promotes GS expression to increase cell proliferation capacity [43, 44]. MYC overexpression is beneficial for GLN utilization as it transforms the substrate of the tricarboxylic acid cycle from glucose to GLN [41]. However, it promotes the utilization of GLN as an amino acid substrate by stimulating the synthesis of asparagine and other amino acids [45]. In addition, MYC promotes GLN uptake by transactivating the GLN transporters SLC1A5 and SLC7A5/SLC3A2. Moreover, it suppresses the transcription of GLS repressor microRNAs-23a/b to increase the expression of GLS, thereby promoting GLN utilization [42, 46, 47]. Furthermore, three other key enzymes involved in GLN utilization are phosphoribosylpyrophosphate (PRPP) amidotransferase, carbamoyl phosphate synthetase II, and CTP synthetase, which are all directly modulated by MYC at the transcriptional level [48]. Interestingly, Wise et al. found that although PI3K/AKT and MYC both regulate the uptake of GLN, the former is not a necessary factor in the process of MYC-mediated GLN uptake [45].

The mammalian target of rapamycin (mTOR) signaling pathway senses changes in the extracellular environment and regulates the collective homeostasis response [49]. It has been previously found that GLN stimulates mTOR signaling. The amino acid pool maintained by GLN in cancer cells is an important factor in the stimulation of mTOR signaling [50]. GLN is the rate-limiting factor in mTOR activation [51]. Cancer cells absorb GLN through SLC1A5 and efflux it through SLC7A5 in exchange for leucine and other essential amino acids (EAAs). The exchanged leucine stimulates mTOR complex 1 (mTORC1) through the Ras-related GTPase (RAG) complex [52]. GLN promotes its own decomposition by binding with leucine, thus activating mTORC, affecting cell growth and autophagy [53]. mTORC1 downregulates SIRT4 by reducing the stability of the proteasome-mediated cAMP response element binding 2 and increasing the expression of glutamate dehydrogenase (GDH), thus resulting in GLN supplementation [54–56]. In addition, SLC1A5 silencing in hepatocellular carcinoma can prevent tumor growth and expansion by inhibiting mTOR signal transmission during the translation process [57].

The acidic TME leads to p53-mediated metabolic reprogramming, which enhances GLN metabolism [51]. GLS2 (liver-type glutaminase) has a different role in distinct cancers. GLS2 regulation by p53 has been confirmed in both non-tumor and tumor cells [58, 59]. The *GLS2* gene contains a *p53* consensus DNA-binding element that promotes interaction between the *GLS2* promoter and the *p53* gene [55]*.* Subsequently, P53 promotes GLS2 expression, both in stress and non-stress conditions, to break down GLN and produce more glutathione and NADH. This results in lowering of intracellular reactive oxygen species (ROS) levels in cells, ultimately targeting energy metabolism and apoptosis [60–62].

**Fig. 3:**
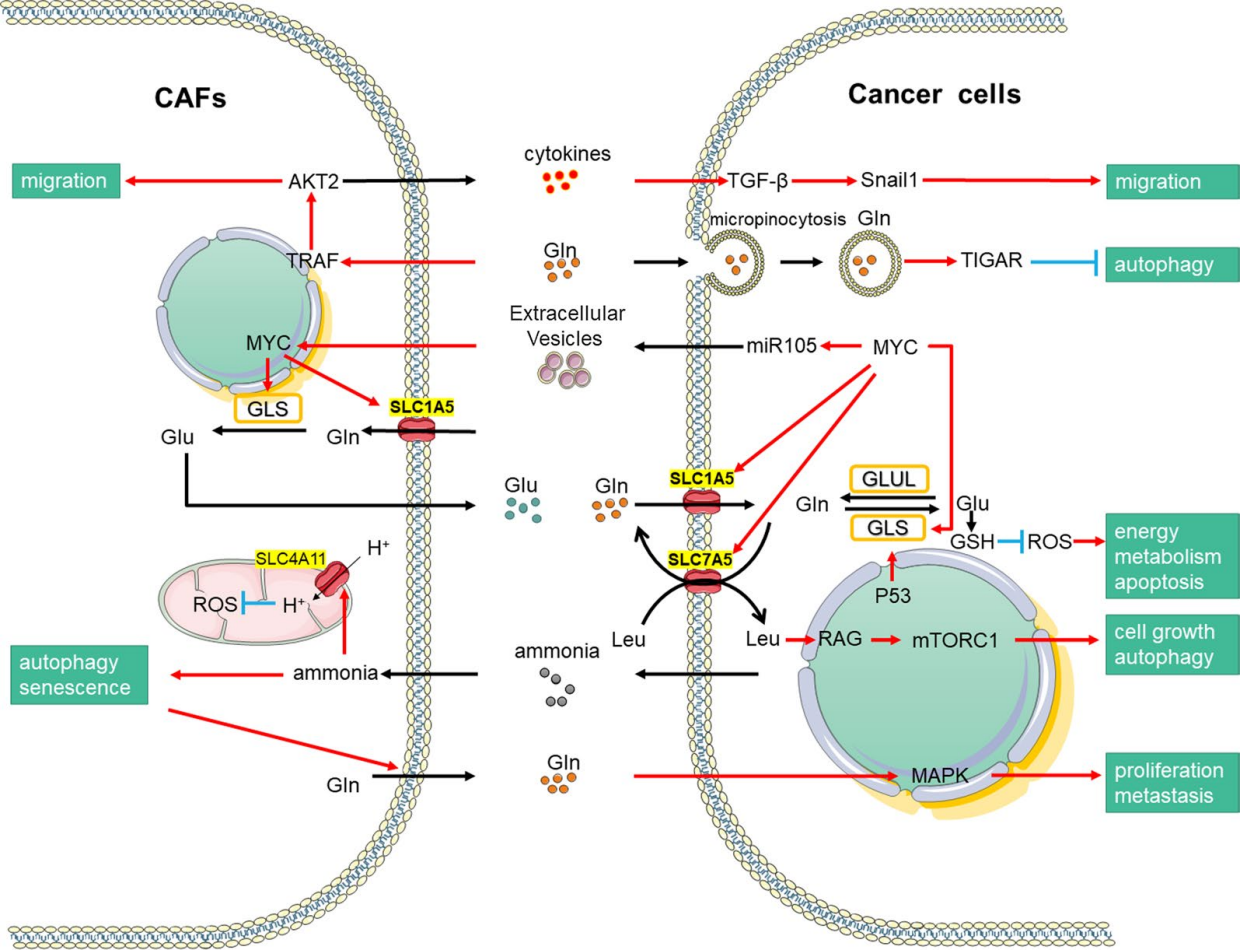
Oncogenic regulation of GLN metabolism. Driven by oncogenes and ammonia concentration, CAFs can secrete nutrients such as Glu and cytokines, thus promoting the metabolic changes of tumor cells and the growth and development of cancer cells. The red pointed arrow heads indicate activation interactions. The black pointed arrow heads indicate material transportation. The blue blunt arrowheads indicate inhibitory interactions

The MAPK pathway is another major player in cancer. Yuan et al. revealed that GLN regulates the mTOR/S6 and MAPK pathways to increase the activities of both GLS and GDH [63]. GLN activates the MAPK pathway to promote proliferation, metastasis, and differentiation of human dental pulp cells [64]. Contrastingly, in macrophages, the MEK/ERK pathway is not a regulator of GLN metabolism [65, 66]. MAPK plays different roles in GLN metabolism in various cell types, which may be because of the unique responses of different types of cells at various nutrition levels [64, 67]. Tumor cells have more potential to proliferate when they are rich in nutrients, whereas immune cells do not show major changes; this is because of the differing metabolic characteristics of the cell types [68]. Therefore, in GLN research, we should consider the functional differences among cell types under physiological conditions, before studying the metabolism differences for GLN (Fig. 3).

## Role of ammonia in cancer

Ammonia is another metabolite that is produced when GLN is decomposed by GS. In earlier studies, ammonia was found to be a toxic metabolic waste product of nitrogenous compounds, such as GLN [69–71]. However, ammonia is usually metabolized into urea through the Krebs cycle and excreted from the body. High concentrations of ammonia may cause autophagy in cancer cells [16].

However, Spinelli et al. reported that growth inhibition of breast cancer cells owing to a GLS inhibitor ceased when ammonia was artificially added [7]. As previously reported, ammonia supports human liver cancer cell growth in GLN-free media [72]. Physiological concentrations of ammonia in the plasma of healthy human adults range from 0 to 50 μM. However, it can be present at up to 150 μM in patients with hyperammonemia [71]. When researchers altered ammonia concentrations in breast cancer cells from 0 to 1 mM, the uptake of GLN or glucose and the expression of GS, GDH, and carbamoyl phosphate synthetase I (CPS1) ammonia-assimilating enzymes did not change [71], indicating that supraphysiological concentrations of ammonia may not interfere with the growth of breast cancer cells; however, they may stimulate breast cancer proliferation and growth and conversion to biomass [7]. Conversely, a study on colon cancer showed that accumulation of ammonia to a concentration of 10 mM downregulates the biosynthesis of polyamine and reduces the proliferation of cancer cells. *P53*, which is considered the most frequently mutated gene in human tumors, represses the urea cycle to suppress ureagenesis and the elimination of ammonia, thereby inhibiting tumor growth [8]. The difference between the two findings may be owing to the differences in the concentration of ammonia. An appropriate concentration of ammonia can therefore promote the proliferation of cancer cells.

However, it is difficult to replace GLN with ammonia as the only nitrogen source for cancer cell metabolism. Studies by Moreno-Sánchez et al. on a variety of cancers, such as ovarian, colon, breast, and prostate cancers, revealed that a moderate concentration of ammonia (0.1–10 mM) increases the proliferation of cancer cells. However, when GLN was removed from the medium, the growth rate of HeLa and MDA-MB-231 cell lines sharply declined [73]. The addition of NH4Cl (1–10 mM) could not rescue these two cell phenotypes. This shows that GLN remains an indispensable nutrient for cancer progression with a strong metastatic ability. Unlike tumor cells, fibroblasts are more sensitive to changes in ammonia concentration. When the concentration of ammonia was increased to 1 mM in the medium, it became too toxic for both mouse and human fibroblasts [73, 74].

The ability of ammonia assimilation in tumor cell lines with stronger metastatic ability was higher than that in non-metastatic tumors. GDH mRNA and protein expression in metastatic tumors was higher than that in non-metastatic tumors [75, 76]. Research on breast cancer indicated that GDH downregulation suppressed ammonia utilization and addition of carbamoyl phosphate synthetase I and GLN synthetase does not rescue this effect. In addition, cancer cells express more GLS1 (kidney-type glutaminase) and GDH than stromal cells [77, 78]. In another study, ammonia significantly increased the expression of GDH and GLS in metastatic hepatocellular carcinoma Hep3B cancer cells [54]. Thus, the utilization of ammonia in cancer cells is closely related to GDH.

Researchers have shown that an H^+^ channel-like membrane transporter (SLC4A11), which is sensitive to ammonia, is expressed in both cancer cells and fibroblasts. SLC4A11 is located in the inner mitochondrial membrane of fibroblasts and is sensitive to ammonia. This sensitivity enables the enhancement of electron transport chain activity, GLN-dependent oxygen consumption, and regulation of ATP levels by reducing ROS production. In GLN-addicted colon cancer cells, SLC4A11 knockdown downregulated GLN catabolism, ROS production, and cell proliferation [79]. Therefore, ammonia acts as a regulator of mitochondrial oxidative stress and promotes GLN catabolism.

## Function of GLN and ammonia in tumor cell nucleotide synthesis

GLN is a material supplier for N-3 and N-9 of adenine and guanine, which is limited by PRPP amidotransferase, which transfers the amino group to the N-glycosidic bond in purine nucleotides [80].

There are two main methods for the de novo synthesis of pyrimidine. The most common way is to synthesize carbamoyl phosphate with GLN as a raw reagent under catalysis by carbamoyl phosphate synthetase II (CPS2). Aspartate transcarbamylase transfers the carbamoyl group to the amino group of aspartic acid to form carbamoyl aspartate. Carbamyl aspartic acid is dehydrated and cyclized to produce dihydroorotic acid, which is then dehydrogenated to form orotic acid (pyrimidine derivative). Orotic acid interacts with PRPP to produce orotic acid nucleotides, which are decarboxylated to form uridine acid [81].

Using inorganic ammonia as a raw material is another way to produce pyrimidine. CPS1 in the mitochondria is used to catalyze the synthesis of carbamoyl phosphate. In previous research, CPS1 was found to be the initiating enzyme of the urea cycle, which plays a role in the conversion of ammonia ions to the less toxic urea, enabling its excretion through the kidneys [7, 82]. Kim et al. demonstrated that carbamoyl phosphate catalyzed by CPS1 can be used to synthesize pyrimidine. The suppression of CPS1 in lung cancer cells leads to pyrimidine synthesis disorder and inhibits tumor growth and proliferation. Addition of exogenous pyrimidine can reverse this DNA damage and rescue growth; however, the process of inhibiting tumor growth is unrelated to the accumulation of ammonia [82].

## Bidirectional crosstalk between cancer associated fibroblasts and cancer cells in GLN and ammonia metabolic pathways

GLN not only plays an important role in tumor cells but is also a very important component of the TME as a whole. In addition to the uptake and secretion of GLN, the excretion and utilization of ammonia are closely related to various components of the TME, especially CAFs. Here, we review the roles of GLN in the transport between cancer cells and CAFs and the metabolic regulation of GLN as a signaling molecule between cells.

### Transport and metabolism of GLN and ammonia in cancer cells and CAFs

As mentioned earlier, cells depend on exogenous GLN as an N donor. Ammonia, which is metabolized using GLN, also plays an important role in regulating the metabolism of tumor cells and CAFs in the TME.

CAFs are stimulated by the ammonia in the TME that is secreted by cancer cells, triggering the activation of autophagy-related signals. In addition, the ammonia taken up by the CAFs in the TME can reduce mitochondrial viability. Together, these two processes cause CAFs to secrete high levels of GLN into the TME. Subsequently, GLN is taken up by cancer cells and metabolized to α-Ketoglutaric acid (α-KG) via the TCA cycle to ultimately increase mitochondrial activity [9]. Simultaneously, GLN stimulates TIGAR activation and inhibits autophagy in cancer cells. The transportation processes of GLN are different in different cancers. For example, pancreatic ductal adenocarcinoma (PDAC) is a cancer with rich stromal cells and poor blood vessel formation and does not have sufficient GLN supply through the serum [83]. However, PDAC cells use micropinocytosis to swallow extracellular proteins and utilize GLN and other amino acids using lysosomes [83–85]. Another route for cancer cell uptake of GLN from the TME is via extracellular vesicles that are released from neighboring cells. A study by Zhao et al. showed that CAFs-derived exosomes provided exosomal cargo and disrupted mitochondrial oxidative metabolism. CAFs-derived exosomes increase GLN reductive carboxylation for biosynthesis in prostate cancer cells by inhibiting the electron transport chain [56]. At the same time, tumor cells, especially epithelial cancer cells, utilize GLN and convert it to both ammonia and glutamate. Among these, ammonia leads to positive feedback and thus promotes CAFs autophagy and support the entry of GLN and other compounds as raw materials in the metabolic pathway [86]. However, compared with CAFs, cancer cells have a stronger resistance to autophagy induced by elevated ammonia levels [16, 86]. Autophagy in CAFs is more common than that in normal fibroblasts in lung cancer [87]. Moreover, some studies have shown that autophagy and senescence markers are highly expressed in stromal cells in breast and lung cancers. Higher matrix autophagy leads to more severe tumor phenotypes, such as the overexpression of Beclin-1 or the loss of Cav-1 in fibroblasts [47]. Similarly, other studies demonstrated that downregulation of Cav-1 in fibroblasts induced a four-fold increase in tumor size [87–89]. In addition, peroxisome proliferator-activated receptor γ (PPARγ) is an antidiabetic target that has a controversial role in cancer therapy. PPARγ is overexpressed in breast CAFs, which promotes autophagy and senescence by activating the HIF1-α and NF-κB pathways. This subsequently promotes the secretion of GLN and other metabolic materials into the TME to enhance the regulatory potential of cancer cells. However, overexpression of PPARγ in cancer cells also leads to modest inhibition of angiogenesis, which suppresses tumor growth [90].

Under some conditions, the release of GLN is not associated with autophagy. Analyses of gene and protein levels in ovarian cancer stroma have shown that expression levels of enzymes that catalyze the synthesis of intracellular GLN, such as glutamic-oxaloacetic transaminase ½ and branch chain amino acid transaminase 1, are higher in CAFs than in normal fibroblasts (NAFs). Half of the glutamate in CAFs, which is the raw material of GLN, is synthesized by the intracellular GLN metabolic pathway, and the other half is transferred from the TME by transporters. Subsequently, patient-derived CAFs secrete GLN into the TME at a rate of 25 pmol/K cells/h, whereas NAFs do not. Cancer cells can also promote GLN secretion using CAFs. CAFs synthesize GLN at the maximum rate when co-cultured with cancer cells [91]. However, on culturing cancer cells that lack GLN, CAFs synthesize more GLN using asparagine and aspartate as raw materials to restore the proliferation of cancer cells [73]. This GLN synthesis can be suppressed by exogenous GS. However, it cannot be suppressed by a GLS inhibitor or GLS siRNA, or even chloroquine, indicating that CAFs autophagy induced by cancer cells lead to CAFs providing nutrition to cancer cells. In conclusion, the transport of GLN and ammonia between CAFs and cancer cells in the TME can be affected by autophagy [92, 93].

### Decomposition and utilization of GLN and ammonia by tumor fibroblasts are regulated by tumor cells

Breast cancer cells activate MYC in CAFs by secreting exosomal microRNA-105, which induces an increase in GLN and glucose metabolism, thus promoting the utilization of lactic acid and ammonia for detoxification and leading to the sustainable growth of tumors [94]. High MYC expression in cancer cells promotes microRNA-105 secretion, which in turn acts on CAFs to stimulate MYC activation, thus extending the effects of MYC originating from cancer cells. CAFs activated by microRNA-105 significantly increased the catabolism of both GLN and glucose while increasing the expression of *GLS* and *SLC1A5* [94]. The levels of glutamate and lactate increased significantly, resulting in accelerated extracellular acidification. Two-dimensional NMR spectroscopy showed that labeled glutamate and acetate exhibited remarkably increased secretion by CAFs and could be involved in fueling cancer cells. Simultaneously inducing glycolysis and GLN decomposition in CAFs can enhance the metabolic flexibility of cancer cells, increase the use of alternative nutrients, and enhance the survival ability under single nutrient deprivation conditions. Simultaneously microRNA-105 could significantly increase the utilization of ammonia to synthesize GLN; thus, when nutrient levels are low and metabolic byproducts accumulate, these CAFs detoxify by converting metabolic waste products (including lactic acid and ammonia) into energy-rich metabolites [42, 91]. When nutrition is adequate, the CAFs, reprogrammed by microRNA-105, can enhance glucose and GLN metabolism to provide fuel for neighboring cancer cells. In addition, breast cancer cells showed higher metastasis and migration abilities when co-cultured with CAFs. Therefore, microRNA-105-mediated stromal cell metabolic reprogramming promotes the continuous growth of tumors by regulating the shared metabolic environment [94].

To better understand the transmission of GLN and ammonia between tumor cells and CAFs, a study established mathematical models on this metabolic transmission of GLN and ammonia. This research group found that it was better to achieve the effect of decomposing tumor cells and utilize GLN-enriched CAFs to consume part of the ammonia to form a metabolic circuit within 48 h [95]. However, this group only performed mathematical modeling for breast cancer cell lines and did not consider many other factors, such as surrounding endothelial cells and immune cells. In future studies, the mathematical model should be improved in relation to metabolism, thereby reducing the workload in actual experiments. It reduces the tenuous links of the experiment; any conclusions drawn from the model can then be verified with actual experiments. This may be an effective evaluation method in the field of metabolic studies.

### Effects of GLN and ammonia on the movement and transformation of tumor cells and CAFs

GLN can be used as an inducer of tumor metastasis. CAFs perceive the GLN concentration in the environment and actively metastasize to high GLN regions while secreting proteases and cytokines to promote the migration of cancer cells behind CAFs as helpers. Roberts et al. found that cancer cells cultured in low GLN were far more tolerant than CAFs. GLN level at the center of breast cancer tumors was lower than in the peripheral area [96]. By simulating the GLN distribution gradient in breast cancer cells, CAFs were found to transfer from low-GLN areas to high-GLN areas, i.e., CAFs tended to migrate to the periphery of the tumor. When receiving the low GLN signature, CAFs activate TRAF, a ubiquitin ligase regulating AKT pathway [97]. TRAF regulates the polarized distribution of AKT2 with the concentration of GLN, which may then lead to the directional migration of CAFs. CAFs also drive cancer cell migration by stimulating the TGF-β-Snail1 axis, which is activated by cytokines derived from CAFs. Cancer cells do not migrate to the high GLN area when cultured alone; however, when co-cultured with CAFs, cancer cells begin to migrate, indicating that CAFs and GLN play a significant role in breast cancer metastasis [98].

In addition, GLS plays an important role in the phenotypic changes in the endothelial–mesenchymal transition (EMT) of tumor cells and CAFs. TGF-β is a crucial factor that promotes tumor proliferation and growth [99]. Silencing GLS during EMT, induced by TGF-β, can reverse this process. Moreover, GLS overexpression is a key factor for TGF-β-induced CAFs to develop a myofibroblast phenotype. Therefore, GLN metabolism is indispensable in TGF-β-promoting cancer [66, 100, 101].

## Clinical application of GLN and ammonia

### Detection of GLN and ammonia utilization for cancer diagnoses

Fluorodeoxyglucose-positron emission tomography scanning is an imaging technology based on the utilization of glucose, which shows the area utilizing more glucose compared to the surrounding areas, thus indicating the possible cancer focus [102]. However, some cancers cannot be detected by positron emission tomography. The survival of tumor cells inevitably requires a lot of energy, and these cells will have found other ways for energy supply. GLN is a potential alternative fuel, but tumor imaging agents based on GLN are still being developed [103].

### Therapeutic strategies utilizing GLN and ammonia regulation

Consistent with the varied effects of GLN in different tumors, it can have distinct effects in different areas of the human body. Current GLN treatments mainly cover the following aspects: reduction in the plasma GLN concentration, inhibition of GLN uptake by tumor cells, use of GLN analogs to decrease tumor cell metabolism, and inhibition of key GLN metabolism enzymes in tumor cells. l-Asparaginase, an essential chemotherapy component in pediatric acute lymphoblastic leukemia, acts by breaking down GLN; however, in the treatment of adult pancreatic carcinoma, l-asparaginase shows unavoidably serious toxicity [24, 104]. Phenylacetate and benzoate have been approved by the FDA for use in the treatment of patients with congenital urea cycle disorders [105]. The combination injection of sodium phenylacetate and sodium benzoate showed remarkable lowering of plasma ammonium levels, but no advances have been made for its application in cancer therapy [105]. This may be because lowering blood GLN levels may cause serious gastrointestinal side effects [106].

#### Suppression of GLN uptake in cancer cells

One major component that supports cancer cells in the uptake of GLN is SLC1A5. Therefore, downregulation of SCL1A5 can reduce growth and proliferation of cells [28]. SLC1A5 is highly expressed in many types of cancers, such as breast, pancreatic, and colon cancers [107–109]. L-g-glutamyl-p-nitroanilide is an inhibitor of SLC1A5 that leads to GLN starvation in cells and inhibits GLN-dependent mTOR activation, thus suppressing cancer progression [9, 63, 110–112]. Moreover, deletion of SLC1A5 and SLC7A11 in mice does not lead to any obvious phenotypes or death [113, 114]. Therefore, both SLC1A5 and SLC7A11 are potential drug targets for cancer therapy. A few ongoing clinical trials are experimentally targeting the SLC7A11 receptor(NCT03965689) with sulfasalazine, a common drug in the treatment of rheumatic and inflammatory bowel diseases; this therapy has been reported to reduce cell proliferation in mice and several cell lines [115].

Administration of GLN analogs is another way to target the GLN pathway, such as the incorporation of antimetabolites in both DNA and RNA. GLN analogs interfere with the synthesis of both purines and pyrimidines. The GLN analog 6-diazo-5-oxo-L-norleucine (L-DON) showed promising preclinical data on its efficacy as an antimetabolite and as a GLS inhibitor; however, it was unsuitable for clinical use because of its excessive toxicity (neurotoxicity, myelosuppression, nausea, and vomiting) [116]. To enhance the effect of L-DON and reduce toxicity, it was co-administrated with PEGylated GLS. As expected, GLN depletion by the action of GLS resulted in a lower distribution of L-DON, leading to an improved toxicity profile. Despite this progress, this therapeutic approach needs further evaluation [117]. Because of the similarity of GLN metabolism between normal cells and cancer cells, GLN therapy often has unacceptable side effects. Thus, further research on therapeutic options targeting GLN metabolism will have two main strategies: on the one hand identifying the differences in glutamine metabolism between tumor cells and on the other hand, increasing the efficacy and reducing the side effects by dual targeting of cancer cells and CAFs, based on the characteristics of the TME.

Many recent clinical trials have focused on GLS and GDH inhibitors, such as compound 968, CB839, and BPTES. However, fatal side effects have been the major limitation in GLN catabolism therapy. Moreover, compound 968 and BPTES showed inhibitory potential in cell cultures [118]. Although the selective GLS inhibitor, CB839, is under clinical investigation, most compounds stagnated in clinical trial phases 1 and 2 (Table 1). CAFs play important roles in the metabolism of GLN and ammonia [7]. Additionally, animal experiments have shown that a combination therapy targeting GLS in cancer cells and GLUL in CAFs is better than using only a single target. This indicates that a combination therapy targeting CAFs along with an inhibitory drug may be a novel strategy for tumor treatment [91].

**Table 1:** Clinical research of targeting GLS [127]

Mainly targets	ClinicalTrials.gov Identifier	Phase	First posted	Drug	Combination drugs
TNBC, NSCLC, renal cell carcinoma	NCT02071862	1	2014	CB-839 (Telaglenastat)	–
CRC	NCT03263429	1/2	2017	CB-839	Panitumumab and irinotecan
AML	NCT03047993	1/2	2017	CB-839	Azacitidine
Hematological tumors	NCT02071888	1	2014	CB-839	–
Clear cell renal cell carcinoma, melanoma, NSCLC	NCT02771626	1/2	2016	CB-839	Nivolumab
NSCLC, CRC	NCT03965845	1/2	2019	CB-839	Palbociclib
Clear cell renal cell carcinoma, TNBC	NCT03875313	1/2	2019	CB-839	Talazoparib
NSCLC	NCT04265534	2	2020	CB-839	Pembrolizumab
TBNC	NCT03057600	2	2017	CB-839	Paclitaxel-carboplatin
Metastatic prostate cancer	NCT04824937	2	2021	CB-839	–
AML, ALL	NCT02071927	1	2018	CB-839	Azacitidine
Ovarian cancer	NCT03944902	1	2019	CB-839	Niraparib
Advanced renal cell carcinoma, metastatic renal cell carcinoma	NCT03428217	2	2018	CB-839	Cabozantinib
Malignant peripheral nerve sheath tumors	NCT03872427	2	2019	Telaglenastat hydrochloride	–
Leptomeningeal neoplasm, metastatic lung non-small cell carcinoma, metastatic malignant neoplasm in the brain	NCT04250545	1	2020	Telaglenastat hydrochloride	–
Plasma cell myeloma	NCT03798678	1	2019	Telaglenastat hydrochloride	Carfilzomib dexamethasone
Astrocytoma	NCT03528642	1	2019	Telaglenastat hydrochloride	Temozolomide
NSCLC	NCT03831932	1	2019	Telaglenastat hydrochloride	Osimertinib

*TNBC* triple negative breast cancer, *NSCLC* non-small cell lung cancer, *CRC* colorectal carcinoma, *AML* acute myeloid leukemia, *ALL* acute lymphocytic leukemia

#### Blocking ammonia reuse

CPS1 has been a target of several clinical investigations in patients with adrenal disease. Till date, there are no known reports on its application in cancer therapy. Celiktas et al. found that a combination of chemotherapy agents and CPS1 knockdown in vitro greatly reduced cell viability in lung adenocarcinoma [119], suggesting that CPS1 is a new target for cancer therapy and thus emphasizing the need for CPS1 inhibitors. Yao et al. developed a high-throughput screening enzyme assay for CPS1 inhibitor candidates and identified H3B-120 as a promising compound. They further evaluated determined the mechanism by which H3B-120 competes with ATP during synthesizing carbamoyl phosphate synthesis. H3B-120 almost completely inhibited the activity of CPS1 but had no effect on CPS2, aspartyl transcarbamylase, and dihydroorotase, which catalyze the first three steps in the de novo pyrimidine synthetic pathway [120]. Therefore, we believe that further studies on CPS1 inhibitors are warranted.

### Cancer-associated drug resistance via GLN metabolism

Tumor drug resistance is a major challenge in any tumor treatment. Studies have shown that increased drug resistance is generally associated with CAFs [121], partly because of the regulation of GLN metabolism by CAFs, leading to drug resistance [86]. In the treatment of prostate cancer, androgen deprivation therapy (ADT)-induced GLN secretion by CAFs is a major cause of ADT resistance. The uptake of GLN by epithelial cells had a similar effect to that of a positive feedback mechanism. L-GLN increased the expression of SLC1A5 and SCL38A2 in a time-dependent manner, and SLC38A4 was also expressed in a GLN-dependent manner. GLN uptake promoted both *GLS* and *GLS2* mRNA expression after 6 h of stimulation, which promoted the use of GLN by tumor cells while stimulating downstream mTOR signaling to promote tumor progression. In addition, GLN secreted by CAFs induce tumor proliferation and differentiation into a more invasive PCA phenotype, namely PCA neuroendocrine differentiation. GLN secretion is sufficient and necessary for this to occur. Studies have shown that in human prostate cancer tissue, RASAL3 methylation levels in CAFs were much higher than those in NAFs. Ras activated macropinocytosis in CAFs and subsequently increased their GLN secretion. Finally, CAFs significantly increased the levels of aminoamide and glutamic acid in cancer cells. A few Krebs cycle intermediates, such as succinate, fumarate, and malate, were significantly elevated and increased the energy metabolism in cancer cells. The significant increase in the level of GLN-derived aspartic acid in epithelial cells co-cultured with CAFs indicates the abundance of a key oxidized precursor of purines and pyrimidines, which further supports cancer cell proliferation [122]. ADT acts by precisely promoting the increase in GLN secretion caused by the apparent silencing of RASAL3, thus increasing drug resistance [123]. Therefore, prostate cancer treatment that involves controlling the CAF-mediated increase in TME GLN level is a promising tumor intervention method.

Radiotherapy is a routine method of tumor treatment, but studies have found that radiation can induce CAFs and promote the utilization of GLNs [124, 125]. Moreover, the basic properties of α-SMA and collagen did not change significantly after irradiation. However, it caused DNA double-strand breaks, *p53* accumulation, and cell cycle arrest in CAFs. IGF1, a paracrine factor released by radiotherapy-treated CAFs, induces IGF1R/InsR phosphorylation in cancer cells, leading to Akt activation. The activated Akt pathway then stimulates mTOR and p70S6 kinase to participate in protein synthesis and cell growth, inducing the promotion of cancer cell survival. IGF1R signaling stimulates an early increase in glucose uptake and lactate release, followed by a decrease in extracellular GLN; moreover, GLN metabolism and transport genes reduce transcription, which accelerates GLN absorption and utilization, resists the damage caused by radiation, and stimulates cancer cell proliferation and metastasis of. Therefore, some patients who are not sensitive to radiotherapy may be protected by CAFs, which, in turn, promote the utilization of GLN in tumor cells to resist injury [126].

## Conclusion

GLN and ammonia play important roles in both biosynthesis and signal transduction. Accumulating evidence has shown that GLN and ammonia levels are also regulated by CAFs. CAFs not only provide nutrients and stimulation for cancer cells but also provide essential physical support that collectively adapts to the metabolic needs of cancer cells; thus, they participate in tumorigenesis. Hence, the metabolic interplay between CAFs and cancer cells is considered an area of vulnerability for GLS therapy. Therefore, targeting the GLN pathway in cancer is not a new idea for cancer therapy, but dual targeting of cancer cells and CAFs is a more promising way to treat cancer.

Thus, CAFs represent a new opportunity for the study of ammonia metabolism in tumor cells. CAFs, being the largest group of cells in the TME, show a remarkable difference in ammonia sensitivity compared to tumor cells, thus implying that the regulation of inorganic ammonia metabolism in tumors is closely related to CAFs. Although research on ammonia metabolism is increasing, we are only at the tip of the iceberg.

## Data Availability

The datasets used or analysed during the current study are available from the corresponding author on reasonable request.

## Abbreviations

ADT: Androgen deprivation therapy
ALL: Acute lymphocytic leukemia
AML: Acute myeloid leukemia
Arg: Ariginine
α-KG: α-Ketoglutaric acid
CAFs: Cancer-associated fibroblasts
Cit: Citrate
CPS1: Carbamoyl phosphate synthetase I
CPS2: Carbamoyl phosphate synthetase II
CRC: Colorectal carcinoma
EAAs: Essential amino acids
GLN: Glutamine
GS: Glutamine synthetase
TME: Tumor microenvironment
ECM: Extracellular matrix
EMT: Endothelial–mesenchymal transition
GDH: Glutamate dehydrogenase
Glc: Glucose
GLS: Glutaminase
GLS2: Liver-type glutaminase
GLS1: Kidney-type glutaminase
Glu: Glutamate
GLUL: Glutamate ammonia ligase
GSH: Glutathione
Lac: Lactose
L-DON: 6-Diazo-5-oxo-l-norleucine
MAPK: Mitogen-activated protein kinase
mTOR: Mammalian target of rapamycin
mTORC1: MTOR complex 1
NAFs: Normal fibroblasts
NSCLC: Non-small cell lung cancer
Orn: Ornithine
PDAC: Pancreatic ductal adenocarcinoma
PPARγ: Peroxisome proliferator-activated receptor γ
PRPP: Phosphoribosylpyrophosphate
PYR: Pyruvate.
RAG: Ras-related GTPase
ROS: Reactive oxygen species
TNBC: Triple negative breast cancer
